# Phillyrin alleviates Kawasaki disease-induced lung inflammation by inhibiting platelet production through the NLRP3/IL-1β/NF-E2 signaling pathway

**DOI:** 10.1186/s13020-026-01348-6

**Published:** 2026-03-07

**Authors:** Da-Hao Mai, Xiaojie Huang, Minsi Liang, Xiaoyin Yu, Wei Hao, Long-Wei Yang, Jing-Rui Feng, Xiao-Le Ling, Ke Wang, Li Zhu, Yang Chen

**Affiliations:** 1 NMPA Key Laboratory for Research of Traditional Chinese Medicine Syndrome, School of Pharmaceutics, Guangzhou University of Chinese Medicine, Guangzhou, 510006 Guangdong China; 2 NMPA Key Laboratory for Research of Traditional Chinese Medicine Syndrome, Science and Technology Innovation Center, Guangzhou University of Chinese Medicine, Guangzhou, 510006 Guangdong China; 3 Chinese Medicine Guangdong Laboratory, Zhuhai, 519031 Guangdong China

## Abstract

**Background:**

The management of Kawasaki disease (KD)-associated complications, including thrombocytosis-driven lung inflammation, remains a clinical challenge, necessitating novel therapeutic exploration. Phillyrin, a principal bioactive compound from *Forsythia suspensa (Thunb.) Vahl*, has shown therapeutic promise in viral lung inflammation. However, its potential therapeutic effect and mechanism of action in KD-induced lung injury are entirely unexplored.

**Aim of the study:**

This study was designed to explore the therapeutic potential of phillyrin against KD-induced thrombocytosis and lung inflammation and its mechanism of action.

**Materials and methods:**

C57BL/6 mice and NLRP3 knockout mice were intraperitoneal injected with *Lactobacillus casei* cell wall extract (LCWE) to establish models of KD-induced lung inflammation**.** MEG-01 cells were also stimulated with LCWE, and subsequently intervened with phillyrin and NLRP3 inhibitor MCC950. The therapeutic effects and mechanisms of phillyrin werestudied both in vivo and in vitro.

**Results:**

In the LCWE-induced mice lung inflammation model, phillyrin dose-dependently decreased circulating platelet counts and reduced CD61⁺ platelets in lung tissue. Hematological analysis demonstrated that phillyrin administration led to a significant reduction in white blood cell count. Furthermore, histopathological examination revealed attenuated inflammatory infiltration and fewer F4/80⁺ macrophages in lung sections. Bioinformatic and molecular docking analyses indicated a strong association between phillyrin's effects and the NLRP3/IL-1β axis. Mechanistic studies demonstrated that phillyrin suppressed megakaryocyte-derived platelet production by inhibiting NLRP3 inflammasome-mediated IL-1β secretion, which consequently disrupts IL-1β-driven NF-E2 expression and leads to substantial downregulation of NF-E2. Furthermore, the suppressive effects of phillyrin on megakaryocytic platelet generation and pulmonary inflammation were consistently enhanced in NLRP3-knockout mice, as well as in MEG-01 cells following either NLRP3 inactivation or IL-1 receptor antagonist treatment.

**Conclusion:**

Phillyrin significantly attenuated LCWE-induced lung inflammation and suppressed megakaryocyte-derived platelet production by inhibiting the NLRP3/IL-1β axis, thereby impeding IL-1β-mediated NF-E2 expression and subsequent thrombopoiesis. These findings identify Phillyrin as a promising therapeutic candidate for Kawasaki disease by targeting the NLRP3/IL-1β/NF-E2 pathway to ameliorate pathological platelet overproduction and pulmonary complications.

**Graphical abstract:**

Phillyrin significantly alleviated LCWE-induced pulmonary inflammation by suppressing megakaryocyte-derived platelet production. Mechanistically, phillyrin inhibited the activation of the NLRP3 inflammasome, thereby impeding caspase-1 maturation and IL-1β secretion. The blockade of IL-1β binding to its receptor consequently downregulated the expression of NF-E2, a pivotal transcription factor regulating platelet production.
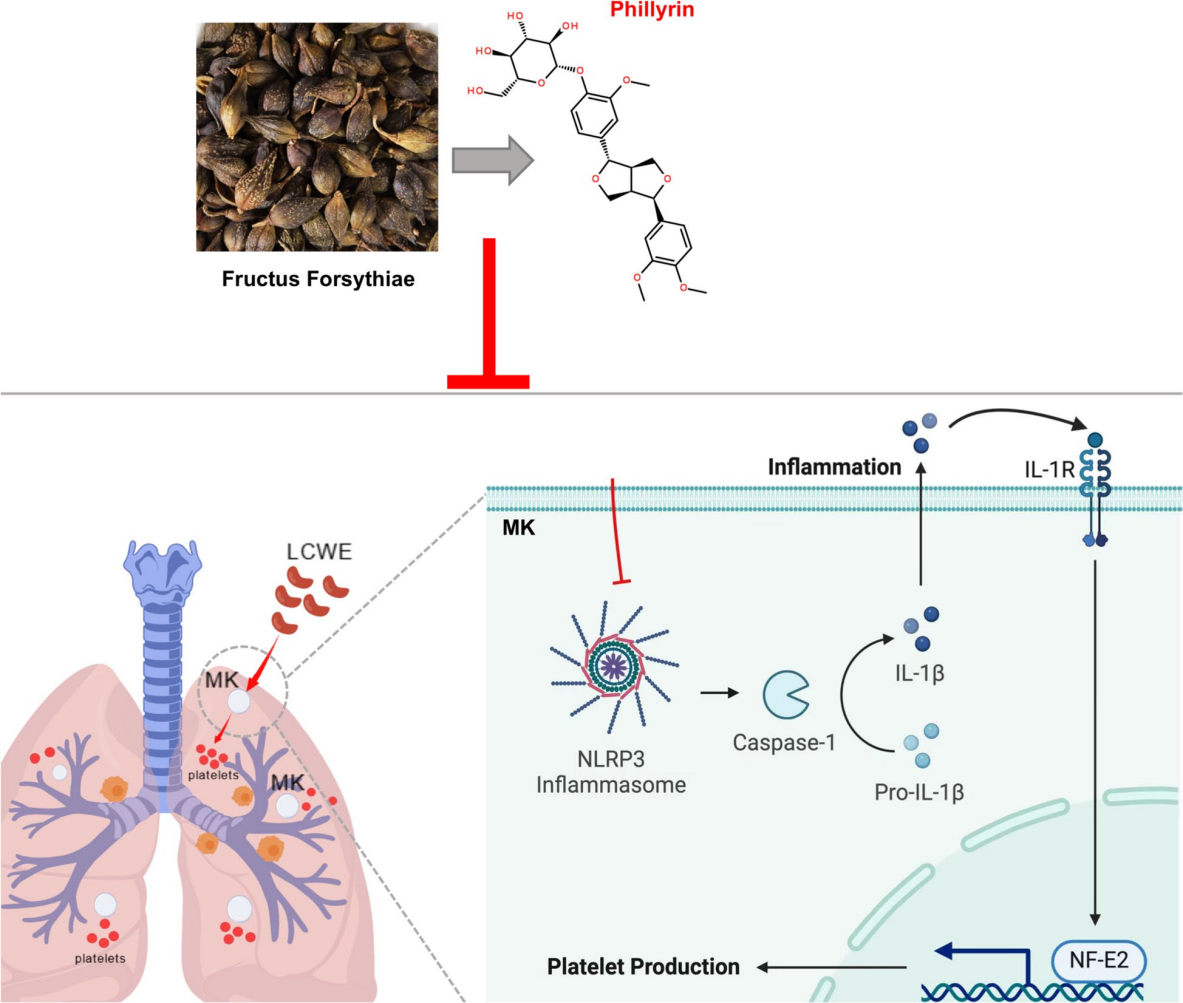

**Supplementary Information:**

The online version contains supplementary material available at 10.1186/s13020-026-01348-6.

## Introduction

Kawasaki disease (KD) is a system vasculitis characterized by increased platelet count and platelet activation [1–3]. Clinical evidences revealed that lung inflammation is one of the most common features in KD patients. KD patients experienced concurrent infections, particularly manifesting as pulmonary symptoms [4]. Moreover, previous studies showed that the chest radiography of KD patients indicated pleural effusion and inflammatory infiltration around peribronchial and interstitial tissue [5–7]. Furthermore, the autopsy data of patients with acute KD also showed signs of pulmonary inflammation [8]. Notably, numerous studies demonstrated the crucial role of platelets in the occurrence and development of pneumonia [9]. A previous study showed that depletion of platelets led to alleviation of pulmonary inflammation [10]. Therefore, inhibition of platelet production is a critical approach to alleviate KD-induced lung inflammation.

Megakaryocytes (MKs) are the cell source of circulating platelets [11, 12] and recent studies revealed MKs also reside in the lung and contributed to platelets production [13, 14]. Furthermore, evidence indicates that inflammatory cytokines, such as IL-1β and IL-6, stimulate platelet production from megakaryocytes (MKs) under inflammatory conditions by upregulating key transcriptional factors, including GATA-1 and NF-E2, which promote MKs maturation and thrombopoiesis. [15, 16]. NLRP3 inflammasome is essential for innate immune system that activates caspase-1 (Casp1) and contributes to the secretion of IL-1β and IL-18 [17]. Accumulating evidence from both clinical and preclinical studies has confirmed that NLRP3 inflammasome activation is a pivotal pathogenic mechanism in Kawasaki disease (KD) and concomitant pulmonary inflammation [18, 19]. However, the precise mechanistic details of how the NLRP3 inflammasome drives platelet production from megakaryocytes (MKs) in the lung by promoting inflammatory cytokine maturation remain largely undefined.

Phillyrin, a bioactive conpounds isolated from *Forsythia suspensa (Thunb.) Vahl*, possesses notable anti-inflammation, antimicrobial, and detoxifying properties, exerted therapeutic potential in inhibiting pulmonary inflammation and platelet activation via inhibiting the activation of NLRP3 [20–24]. Based on this, we hypothesized that phillyrin alleviates lung injury by suppressing platelet production via the NLRP3/IL-1β signaling pathway. However, the underlying mechanism requires further elucidation.

Hence, in this study, we dedicated to investigate the effect and potential mechanism of phillyrin on KD-induced platelets production and lung inflammation based on NLRP3/IL-1β axis.

## Materials and methods

### Preparation of LCWE

L.casei bacteria (ATCC 11578, American Type Culture Collection, VA) was maintained in Lactobacillus MRS broth (BD system, USA) on a shaker platform (37 °C). Bacterial cultures were collected at mid-logarithmic phase through centrifugation (10,000×*g*, 40 min), followed by six successive phosphate-buffered saline (PBS, pH 7.4) rinses. Cellular disruption was achieved through overnight incubation (ambient temperature, orbital shaking) with 4% sodium dodecyl sulfate (SDS; MilliporeSigma, MO), succeeded by eight additional PBS purification cycles. The lysate underwent sequential enzymatic digestions with: (1) RNase (250 μg/mL); (2) DNase (250 μg/mL); (3) Trypsin–EDTA (250 μg/mL) (Sigma-Aldrich, MO). Each incubation was followed by 2 washes in PBS and then 4 washes only after an incubation with trypsin. The processed biomass (3 g wet weight) was reconstituted in 9 mL PBS and subjected to cryogenic sonication (Dry ice/ethanol bath, 2 h) using a tuned probe assembly (1/8″ microtip, 20 kHz frequency; Fisher Scientific, Nepean, Canada) operating at 50% duty cycle (10 s pulse/5 s interval). Clarified supernatant obtained after ultracentrifugation (1 h, 4 °C) constituted the L. casei whole-cell extract (LCWE). Quantification employed the phenol–sulfuric acid method with rhamnose as the standard, with final concentrations expressed as mg equivalent/mL PBS.

### Animal experiment

All experimental protocols were conducted under ethical approval (License No. xs20240160) granted by the Institutional Animal Care and Use Committee at Guangzhou University of Chinese Medicine, China. Male C57BL/6 mice (5-week-old) were acclimatized in specific pathogen-free conditions with controlled ambient temperature under 12/12-h photoperiod cycles, provided free access to standard chow and autoclaved water. Experimental cohorts received intraperitoneal administration of either: (1) 0.5 mg Lactobacillus casei whole-cell extract (LCWE); (2) Equivalent volume phosphate-buffered saline (PBS; vehicle control); Subsequent therapeutic interventions (initiated 24 h post-challenge) included daily i.p. injections for 7 consecutive days of: (1) Dexamethasone (5 mg/kg); (2) Phillyrin (10, 20, or 40 mg/kg body weight/day). A randomized block design allocated subjects into six experimental groups (n = 10/group): Control group (PBS-treated); LCWE group; Dexamethasone treatment group (5 mg/kg); Phillyrin low-dose group (10 mg/kg); Phillyrin medium-dose (20 mg/kg); Phillyrin high-dose (40 mg/kg).

### Immunohistochemistry

Following dewaxing, rehydration, and antigen retrieval performed using 0.01 M citrate buffer (pH 6.0), 4-μm-thick lung paraffin sections underwent sequential processing. Tissue sections were initially treated with 3% hydrogen peroxide for 10 min to quench endogenous peroxidase activity. Non-specific binding sites were subsequently blocked through incubation with 5% normal goat serum. Primary antibody immunostaining was conducted using F4/80 antibody (1:200 dilution; Santa Cruz Biotechnology, USA) in an overnight incubation at 4 °C. The detection system involved sequential 30-min incubations with species-matched biotinylated secondary antibodies (1:200; Vector Laboratories, USA) and horseradish peroxidase (HRP)-conjugated streptavidin (1:200; Boster Biological Technology, China). Chromogenic development was achieved by 2-min exposure to 3,3'-diaminobenzidine (DAB) substrate (Vector Laboratories, USA). Cellular nuclei were visualized through hematoxylin counterstaining. Following a final permeabilization step, permanent mounting was accomplished using commercial mounting medium to preserve histological integrity.

### Blood cell analysis

The plasma preparation method is the same as *2.3*. The blood cell analyzer (Mindray, Shenzhen, China) is used to detect the shape, size and distribution of blood cells.

### Blood smear microscopy

The morphology, size and distribution of platelets was observed by staining with Wright's solution on blood smear. After blood collection of capillary orbital venous plexus, a glass rod was used to add 1 drop of anticoagulant blood 1 cm away from one end of the slide, and the slide was held flat in the left hand to push the blood into a blood smear with appropriate thickness. Then drop the Reith dye on the blood film until the dye is submerged in the whole blood film, and dye for 1 min. Add an equal amount of distilled water and mix with the dye solution for 5 min. Finally, rinse the dye with distilled water, blot with absorbent paper, and observe after natural drying.

### Hematoxylin–eosin (H&E) staining

Excised pulmonary tissue specimens underwent immediate immersion in neutral buffered 4% paraformaldehyde solution (PFA; pH 7.4) to achieve protein denaturation and structural stabilization. Tissues were subsequently subjected to progressive dehydration through an ascending ethanol series, followed by xylene clearing and paraffin infiltration using standard histological protocols. Cells were stained by H&E method according to the procedure.

### ELISA assay

Interleukin 1 beta (IL-1β) in bronchoalveolar lavage fluid was detected using ELISA kit (MEIMIAN, China) and test according to manufacturer's instructions.

### Cell culture

The MEG-01 cells are derived from human megakaryocytic leukemia cell line and obtained from NCM Biotech (Suzhou, China). MEG-01 cells were maintained in RPMI 1640 supplemented with 10% Fetal Bovine Serum (FBS) and 1% penicillin/streptomycin solution (P/S). The cells were incubated with 5% CO_2_ at 37℃. The cells were divided into six groups: control group, model group with LCWE (10 μg/mL), different dose of Phillyrin group (15.625, 31.25, 62.5 μg/mL) and Dexamethasone group (5 μM) and then incubated for 24 h. LCWE were administrated with phillyrin or dexamethasone simultaneously.

### May-Grünwald stain and Giemsa stain

MEG-01 cellular specimens were fixed in chilled methanol (5 min) followed by deionized water rinsing. Sequential staining protocols were implemented: May-Grünwald solution (1:1 aqueous dilution, 5 min) was directly replaced with Giemsa working solution (1:9 aqueous dilution, 30 min) without intermediate rinsing. Post-staining procedures included triple deionized water washes prior to microscopic examination.

### Immunofluorescence

Cellular specimens underwent fixation in 4% paraformaldehyde (PFA) for 20 min, followed by three sequential phosphate-buffered saline (PBS) rinses. Primary antibody incubation was conducted at 4 °C for 16–18 h. Following PBS washes, specimens were co-stained with species-matched secondary antibodies conjugated to Alexa Fluor 488/555 fluorophores (Thermo Fisher Scientific, USA) for 2 h under ambient conditions. Fluorescence imaging was performed using a LSM 880 confocal microscope (Zeiss, Germany), with colocalization parameters quantified through Image-Pro Plus software (Media Cybernetics, USA).

### Western blotting

Intracellular protein was extracted conventionally, equal amounts of the protein samples were separated by 12% SDS-PAGE and transferred onto 0.2 μm polyvinylidene fluoride membranes. Then, 5% skim milk powder was enclosed at room temperature for 2 h. The blocked membrane was incubated with the indicated primary antibodies at 4 °C overnight and then treated with anti-rabbit IgG (1:1500; CST) or anti-mouse IgG (1:1500; CST) for 2 h at room temperature. The anti-β-actin (1:1000; CST) was used as an internal control. Proteins expressions were analyzed by using ImageJ.

### FCM (flow cytometry)

MEG-01 cells were collected by centrifugation at 1000 rpm per minute. Then incubated for half hour with primary antibodies at room temperature. The tubes were washed and labeled with corresponding Alexa Fluor- 488, Alexa Fluor-594 conjugated secondary antibodies (Invitrogen, Carlsbad, CA, United States) co-incubated for half hour at room temperature.

### Network pharmacology research

A total number of 434 thrombocytosis-, 1052 genes vasculitis-, and 202 pneumonia-related gene, applying a relevance score threshold (R > 1) for inclusion from the Genecards databases (https://www.genecards.org/). In addition, Venn maps of vasculitis-associated, pneumonia-associated targets and thrombocytosis- associated targets were studied, revealing the overlapping targets among the 3 gene sets. Functional enrichment studies of the core targets were carried out based on David database (https://david.ncifcrf.gov/).

### Molecular docking

To get Protein and ligand structure file: PubChem (http://pubchem.ncbi.nlm.nih.gov/) and Protein Data Bank (http://www.rcsb.org/pdb) in the database search phillyrin SDF Structure file and 3D crystal structure of NLRP1, AIM2, NLRP3 and NLCR4, and save file format suffix.pdb. Small molecules are introduced into ChemBio3D Ultra 21.0.0.28 for energy minimization. The optimized small molecules were imported into AutodockTools-1.5.6 for hydrogenation, charge calculation, charge distribution, etc. The protein was introduced into Pymol2.3.0 to remove the protein crystal water and the original ligand, and the protein structure was introduced into AutoDocktools (v1.5.6) for hydrogenation, charge calculation, charge distribution, etc. AutoDock Vina1.1.2 is used for interconnection.

### Statistical analysis

The data were statistically analyzed using Graph-Pad Prism8.0. Data were represented as the means ± Standard Deviation (SD) of at least three independent experiments in vitro or six mice of each group in vivo. Statistical differences of means were assessed by using one-way analysis of variance (ANOVA) and post hoc tests in the independent variable with more than 2 groups.* P* values less than 0.05 (*P* < 0.05) were considered statistically significant.

## Results

### Phillyrin reduced LCWE-induced platelet production and inflammation in the lung of mice

**Fig. 1:**
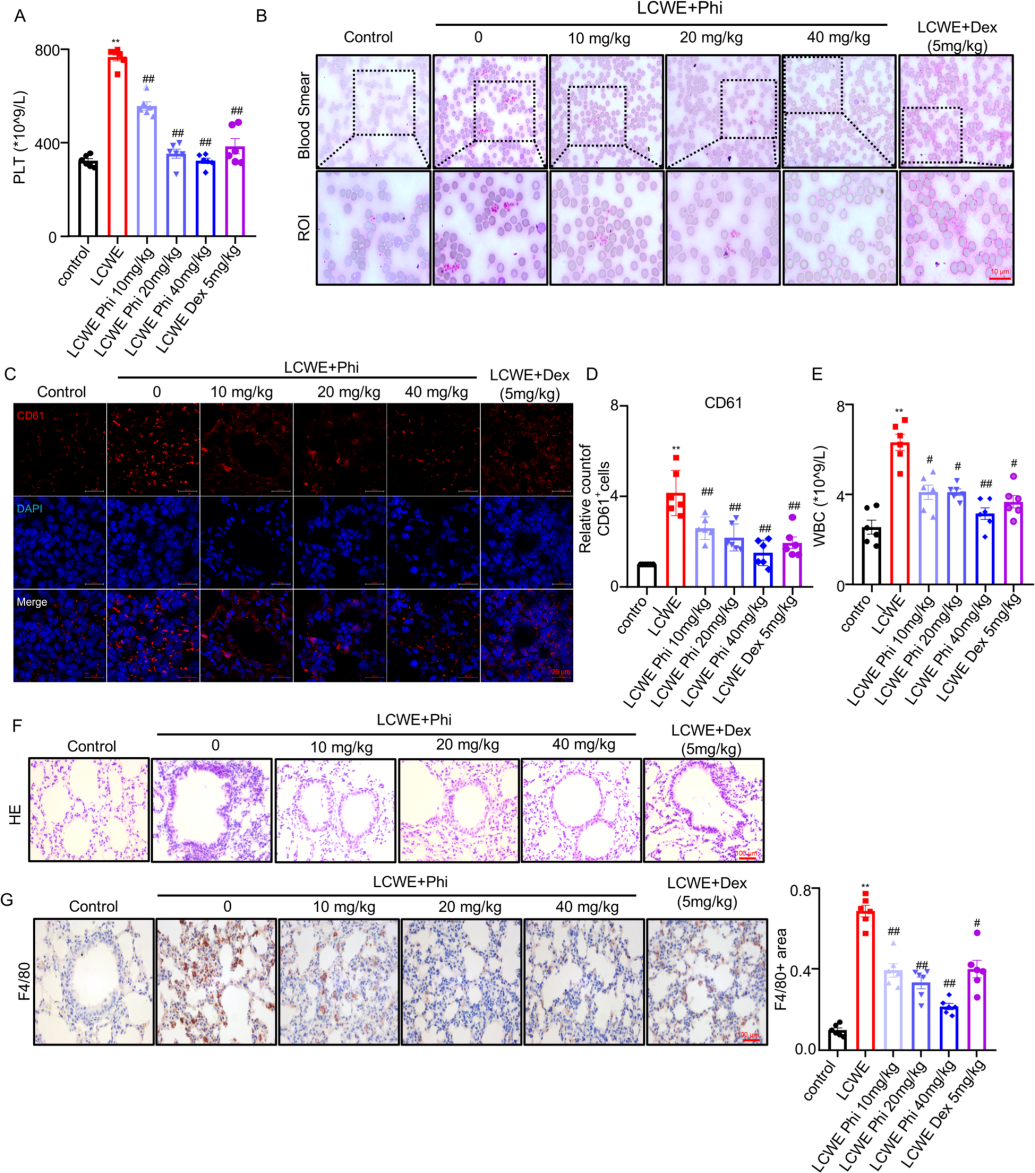
Phillyrin reduced LCWE-induced platelet production and inflammation in the lung of mice. **A** The platelet count (PLT) in the blood; **B** blood smear microscopy, scale bars, 20 μm; **C, D** representative confocal images and quantification of CD61 in the lung of mice. CD61(red), DAPI (blue). Scale bars, 20 μm. **E** The WBC count in the blood, **F** representative image of H&E staining, scale bar, 100 μm. **G** Typical image of immunohistochemistry staining and quantification of F4/80 in the lung, scale bar: 100 μm. n = 6 per group. Data are presented as mean ± SD. *P < 0.05 versus Control group; **P < 0.01 versus control group, ^#^*P* < 0.05; ^##^*P* < 0.01 versus LCWE group

A mouse model of Kawasaki disease (KD) was established by intraperitoneal injection of LCWE. Successful model induction was confirmed by detecting the expression level of vascular endothelial VCAM-1 via immunofluorescence (Fig. S1A–S1B). To assess the effect of phillyrin on platelets in KD mice, platelet levels were measured on day 7 post-LCWE injection. Results showed that phillyrin dose-dependently attenuated LCWE-induced elevation of PLT (Fig. 1A). Peripheral blood smear analysis demonstrated that phillyrin dose-dependently reduced platelet aggregation (Fig. 1B). Consistent with this finding, immunofluorescence quantification of pulmonary tissues revealed that phillyrin significantly attenuated the LCWE-induced accumulation of platelets in the lungs in a dose-dependent manner (Fig. 1C, D). Accumulation of platelets is a crucial pathogenic factor of lung inflammation. Hence, the counts of white blood cell (WBC) and pulmonary F4/80 level were evaluated. Serum white blood cell (WBC) levels were markedly increased in mice following LCWE induction, whereas phillyrin treatment dose-dependently lowered WBC counts (Fig. 1E). Furthermore, hematoxylin–eosin (H&E) staining and immunohistochemical analysis for the macrophage marker F4/80 revealed that LCWE triggered significant inflammatory cell infiltration in lung tissue, which was ameliorated by phillyrin in a dose-dependent manner (Fig. 1F–G). In addition, the safety profile of phillyrin was assessed in mice treated with doses of 20, 40, 80, and 160 mg/kg for one week. Evaluation of organ-to-body weight ratios and histopathological examination of the lungs, liver, and kidneys showed no significant differences between the treatment groups and the control group, demonstrating that phillyrin administration at these concentrations did not induce significant toxic effects (Fig. S2). Collectively, these data indicated that phillyrin effectively alleviated LCWE-induced pulmonary inflammation and histopathological damage by suppressed the accumulation of plateletin the lungs of mice.

### *Phillyrin inhibited platelet production *via* inhibiting megakaryocytic NLRP3 inflamasome activation in the lung of mice*

**Fig. 2:**
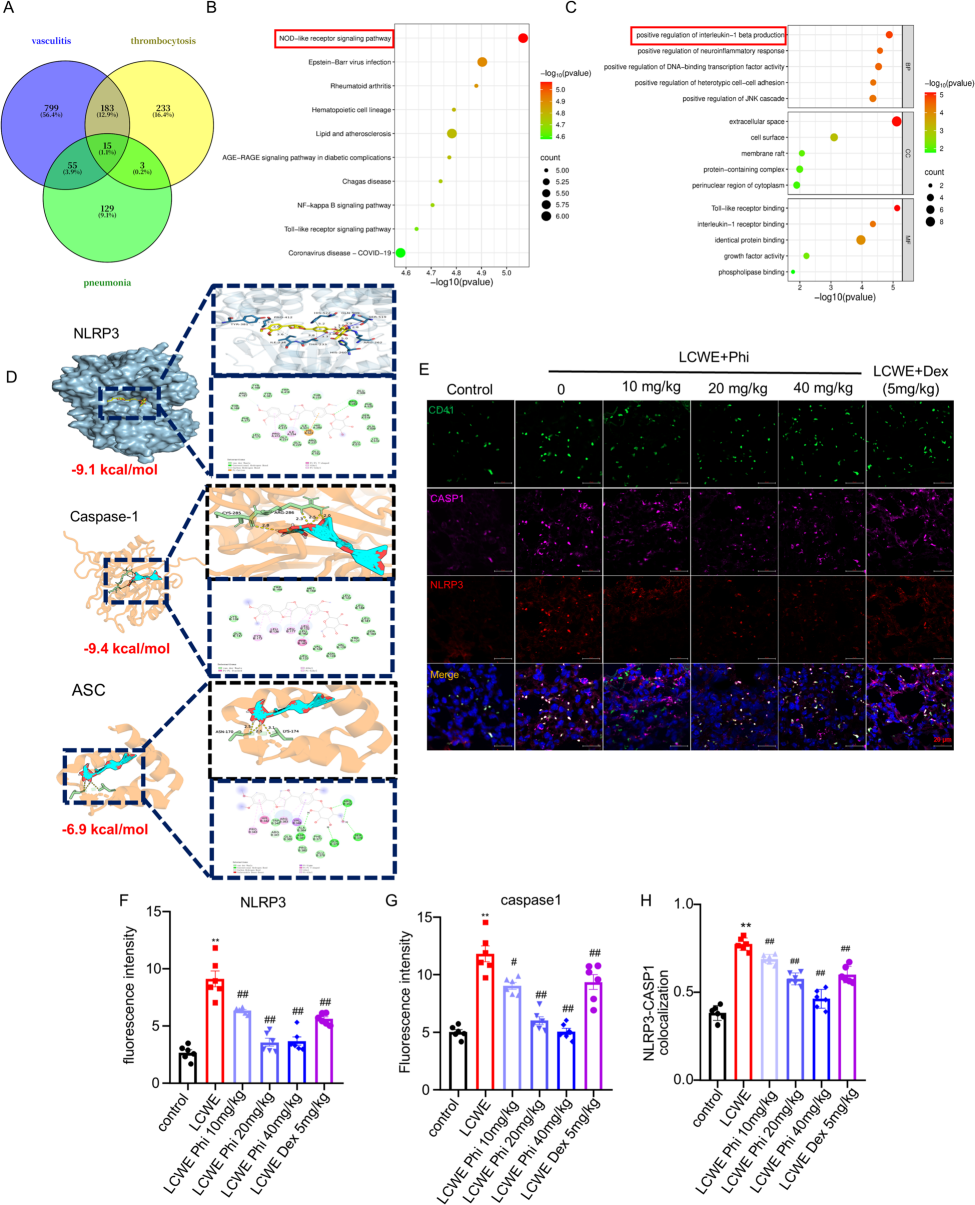
Phillyrin inhibited platelet production via inhibiting megakaryocytic NLRP3 inflammasome activation in the lung of mice.** A** The Venn diagram of the intersection of genes related to pneumonia, thrombocytosis and vasculitis. **B** Gene Ontology (GO) pathway analysis of pneumonia-thrombocytosis-vasculitis intersected genes. **C** KEGG pathway analysis of pneumonia-thrombocytosis-vasculitis intersected genes. **D** 3D Molecular Docking Conformations of Phillyrin with NLRP3, Caspase-1, and ASC Complex. 2D schematic diagrams showed the interaction bond and active sites. Green dashed lines refer to the convention hydrogen bond; orange dashed lines refer to π-cation, light pink dashed lines refer to π-Alky; purple dashed lines refer to π-sigma. **E** Representative confocal images of megakaryocytic Caspase-1, NLRP3 in the lung of mice. CD41(Green) was used to label the megakaryocyte, CASP1(violet), NLRP3 (Red), scale bar: 10 μm. **F–H** Quantification of CASP1, NLRP3 and their colocalization in pulmonary megakaryocyte. n = 6 per group. Data are presented as mean ± SD. **P < 0.0001 versus control group; ^#^*P* < 0.05; ^##^*P* < 0.01 versus LCWE group

To elucidate the mechanism by which phillyrin alleviates pulmonary platelet accumulation in KD, we systematically compiled 434 thrombocytosis-, 1052 genes vasculitis-, and 202 pneumonia-related gene, applying a relevance score threshold (R > 1) for inclusion from the Genecards database. A Venn diagram analysis of these three gene sets was performed, which identified 15 core intersecting genes common to pneumonia, thrombocytosis, and vasculitis (Fig. 2A). Moreover, GO and KEGG analysis of the 15 overlapping genes identified "regulation of IL-1β production" and the "NOD-like receptor signaling pathway" as top enriched terms, suggesting their involvement in the molecular mechanism underlying KD-induced excessive platelet production (Fig. 2B, C). The secretion of IL-1β can be mediated by multiple inflammasomes, including NLRP3, NLRP1, AIM2, and NLRC4. Their expression in response to phillyrin was assessed by qPCR, which revealed that phillyrin exerted the most potent suppression on NLRP3 (Figure S3A). Previous studies revealed NLRP3 inflammasome activation is involved in KD [25]. Thus, molecular docking analysis was performed to assess the binding of phillyrin to the NLRP3 protein, the binding affinity between NLRP3 (PDB ID: 3QF2), caspase-1(PDB ID: 1BMQ), ASC (PDB ID: 5H8O)and phillyrin (PubChem CID: 101712) was assessed using AutoDockVina 1.1.2, the binding energy of -9.1 kcal/mol, -9.4 kcal/mol and -6.9 kcal/mol, respectively. For the NLRP3-phillyrin complex, interactions involved both conventional hydrogen bonds and π interactions with key residues including THR233, GLU306, ARG262, and TYR381, among others. The caspase-1-phillyrin interaction was primarily dominated by π interactions with residues including ARG352, TRP340, HIS342 and so on. Moreover, the ASC-phillyrin complex exhibited both conventional hydrogen bonds and π interactions, involving residues including LEU122, TRP131, LYS139 and others (Fig. 2D). Subsequently, we investigated whether phillyrin ameliorates LCWE-induced thrombocytosis by suppressing NLRP3 inflammasome activation in pulmonary megakaryocytes. Pulmonary megakaryocytes were marked with CD41, NLPR3 activation was detected via immunofluorescence staining. Results showed that LCWE treatment significantly activated the NLRP3 inflammasome in pulmonary megakaryocytes, indicated by upregulated NLRP3 and caspase-1 expression and their increased colocalization. This effect was notably attenuated by phillyrin in a dose-dependent manner (Fig. 2E–H). Together, these data suggested that phyllyrin impeded excessive pulmonary platelets production via inhibiting the activation of pulmonary megakaryocytic NLRP3 inflammasome in mice.

### NLRP3 knockout enhanced the effect of phillyrin on platelet production and inflammation in the lung of mice

**Fig. 3:**
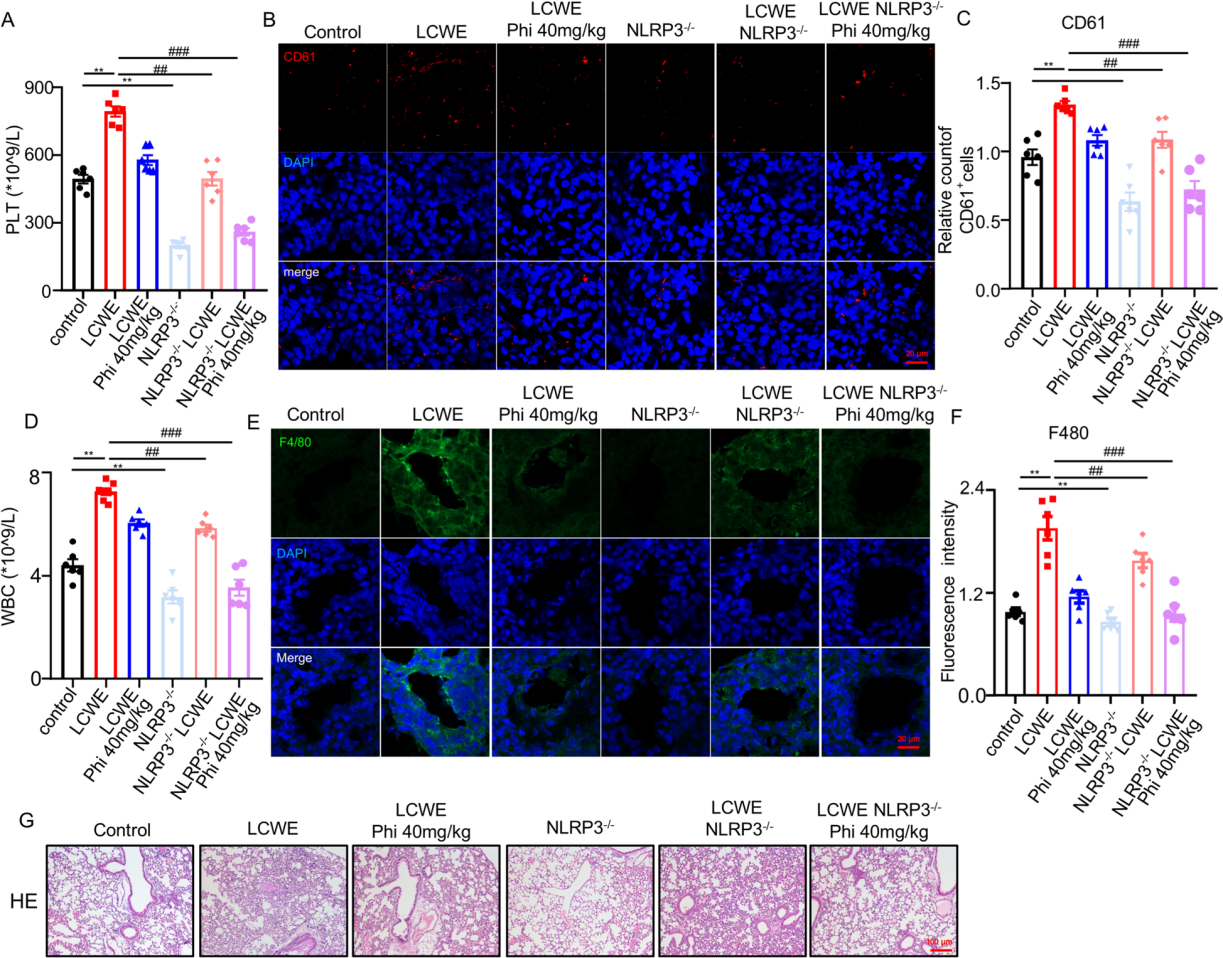
NLRP3 knockout enhanced the effect of phillyrin on platelet production and inflammation in the lung of mice. **A** The platelet count (PLT) in the blood. **B, C** Representative confocal images and quantification of CD61 in the lung of mice. CD61 (red), DAPI (blue). Scale bars, 20 μm. **D** The WBC count in the blood. **E, F** Representative confocal images and quantification of F4/80 in the lung of mice. F4/80 (green), DAPI (blue). Scale bars, 20 μm. **G** Representative image of H&E staining, scale bar, 100 μm. n = 6 per group. Data are presented as mean ± SD. *P < 0.05, **P < 0.01, versus Control group; ^#^*P* < 0.05,^##^*P* < 0.01, ^###^*P* < 0.001 versus LCWE group

To further validate interaction between NLRP3 signalling pathway and the effect of phillyrin on platelet production, NLRP3 knockout (NLRP3-KO) mice were employed. The NLRP3-knockout mouse was generated by targeting essential functional domains of the Nlrp3 gene, thereby ablating its activation capacity. To validate the knockout efficiency in lung tissue, we assessed the levels of cleaved caspase-1 and cleaved IL-1β. The results confirmed that NLRP3 knockout contributed to a pronounced reduction in the production of cleaved caspase-1 (decreased by 66.7%) and cleaved IL-1β (decreased by 89.7%) compared to wild-type controls (Figure S3B, 3C).NLRP3-KO mice exhibited significantly lower platelet counts in both blood and lungs. Crucially, the absence of NLRP3 synergistically enhanced phillyrin's efficacy in reducing platelet accumulation (Fig. 3A–C). Consistent with these results, NLRP3 knockout potently enhanced the inhibitory effect of phillyrin on WBC counts (Fig. 3D). Furthermore, immunohistochemical analysis for the macrophage marker F4/80 and H&E staining also revealed that NLRP3 deficiency enhanced the therapeutical effects on inflammatory infiltration in the lung (Fig. 3E–G). Together, these data further confirmed that phillyrin exerts therapeutic effects on LCWE-induced pulmonary thrombocytosis and inflammation by inhibiting the activation of NLRP3 inflammasome.

### Phillyrin reduced platelet production in MEG-01 cells

**Fig. 4:**
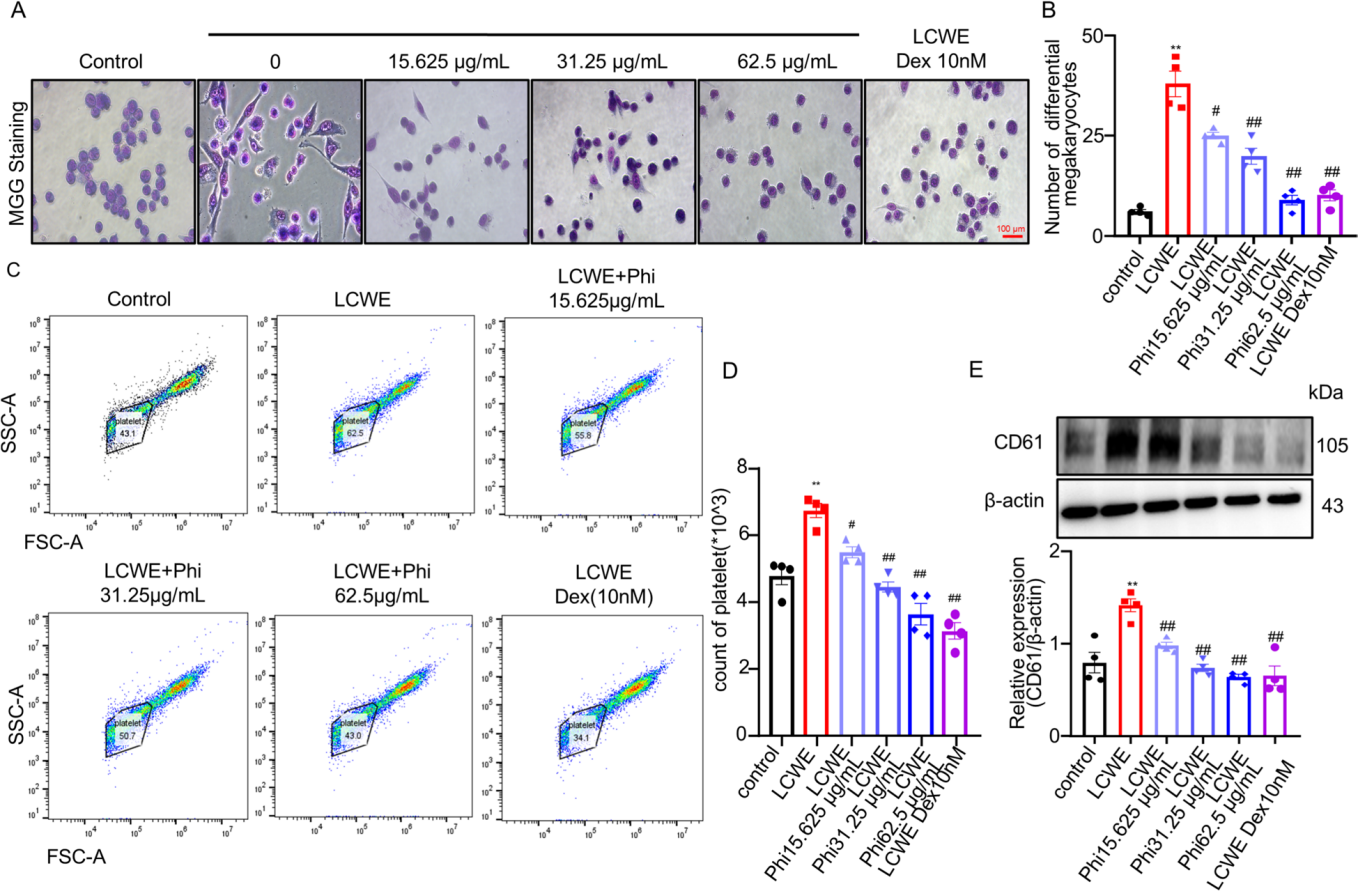
Phillyrin reduced platelet production in MEG-01 cells. **A** Representative image of MGG staining in MEG-01cells. **B** Representative image of MGG stain and count of differential megakaryocytes. **C, D** Representative flow cytometric images and quantification of platlet count in MEG-01cell. **E** The protein expression of CD61 in MEG-01cells. n = 4 per group. Data are presented as mean ± SD. *P < 0.05 versus Control group, **P < 0.01, versus Control group; ^#^*P* < 0.05, ^##^*P* < 0.01, ^###^*P* < 0.001 versus LCWE group

To further validate the effects of phillyrin on platelet production, in vitro experiments were performed using MEG-01 cells. In vitro cytotoxicity was assessed in MEG-01 cells using the CCK-8 and LDH release assays. Treatment with phillyrin at concentrations ranging from 15.625 to 125 μg/mL did not induce significant cell death compared to the control group (Figure S3D, 3E). Subsequently, May-Grünwald-Giemsa (MGG) staining revealed that LCWE stimulation triggered megakaryocytic differentiation in MEG-01 cells, as evidenced by pseudopodia formation, which was markedly suppressed by phillyrin treatment (Fig. 4A, B). Moreover, flow cytometry (FACS) analysis demonstrated a significant increase in platelet count following LCWE treatment, and this effect that was reversed by phillyrin in a dose-dependent manner (Fig. 4C, D). Furthermore, immunoblotting of MEG-01 cell culture supernatant confirmed that the upregulation of CD61, a platelet-specific marker, induced by LCWE was subsequently downregulated by phillyrin (Fig. 4E, F). Together, these data indicated phillyrin effectively inhibits LCWE-induced megakaryocytic differentiation and platelet production.

### *Phillyrin inhibited platelet production *via* inhibiting NLRP3/IL-1β axis in MEG-01 cells*

**Fig. 5:**
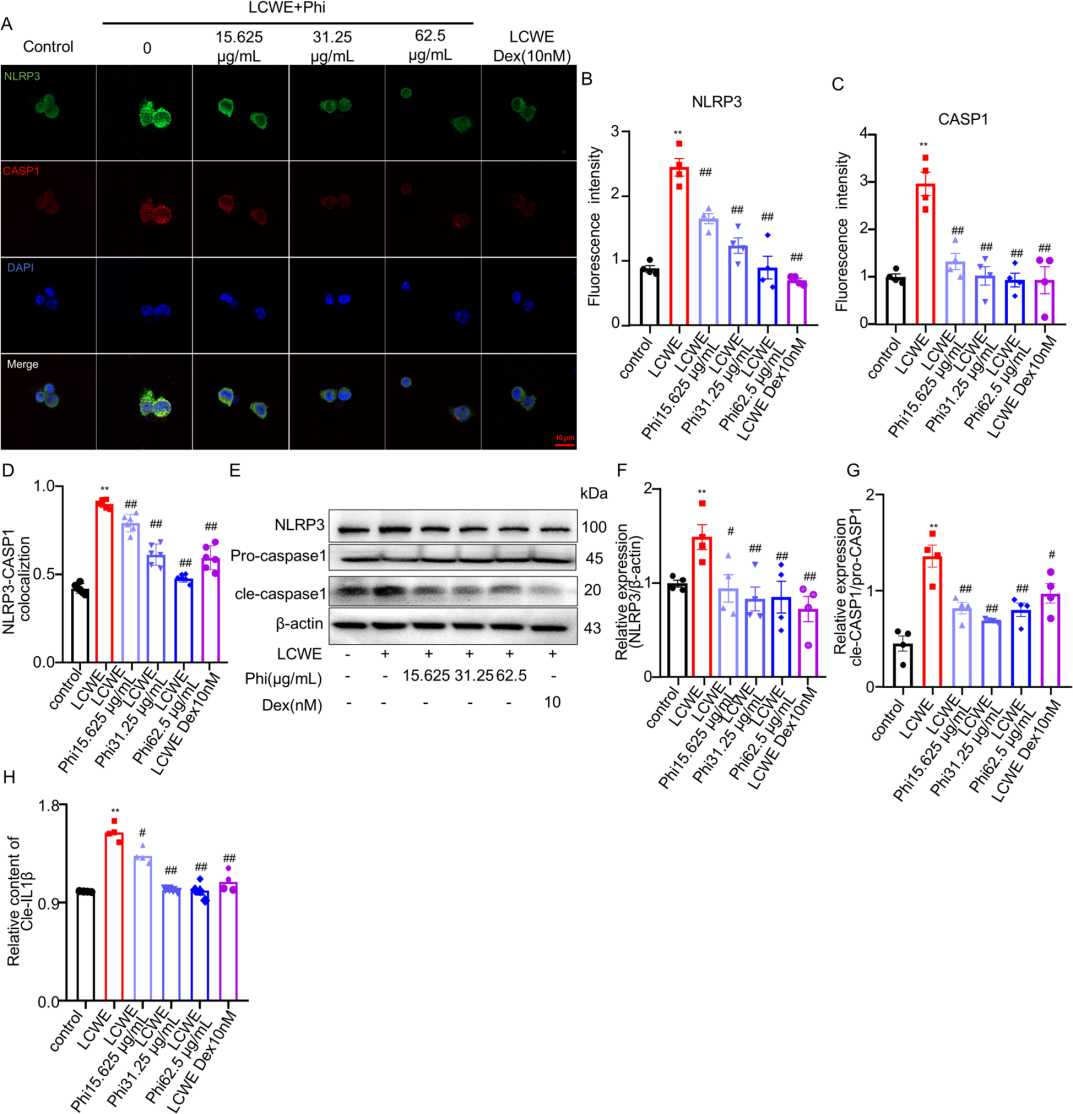
Phillyrin inhibited platelet production via inhibiting megakaryocytic NLRP3/IL-1β axis in MEG-01 cells. **A** Representative confocal images of CASP1, NLRP3 in MEG-01 cells. NLRP3 (green), CASP1 (red), DAPI (blue), scale bar: 10 μm. **B–D** Quantification of CASP1, NLRP3 and their colocalization in MEG-01 cells. **E–G** Immunblot analysis of NLRP3, pro-caspase1 and cle-caspase1 level in MEG-01 cells. **H** ELISA results of secreted of IL-1β. n = 4 per group. Data are presented as mean ± SD. **P < 0.01 compared with control group. ^##^*P* < 0.01, versus LCWE group

To further elucidate whether phillyrin suppresses platelet production by inhibiting NLRP3 inflammasome activation in MEG-01 cells, we assessed NLRP3 and caspase-1 colocalization via immunofluorescence. Phillyrin treatment dose-dependently reduced both the protein levels of NLRP3 and caspase-1 and their colocalization compared to the LCWE group (Fig. 5A–D). Consistent with these findings, immunoblot analysis confirmed that phillyrin significantly suppressed the LCWE-induced upregulation of NLRP3 and cleaved caspase-1 in cell lysates (Fig. 5E–G). Furthermore, the marked increase in secreted IL-1β concentration induced by LCWE, as measured by ELISA, was also abolished by phillyrin (Fig. 5H). These findings indicated that phillyrin inhibits platelet generation in MEG-01 cells by blocking the NLRP3/IL-1β signaling pathway.

### Inhibition of NLRP3 enhanced the effect of phillyrin on platelet production in MEG-01 cells

**Fig. 6:**
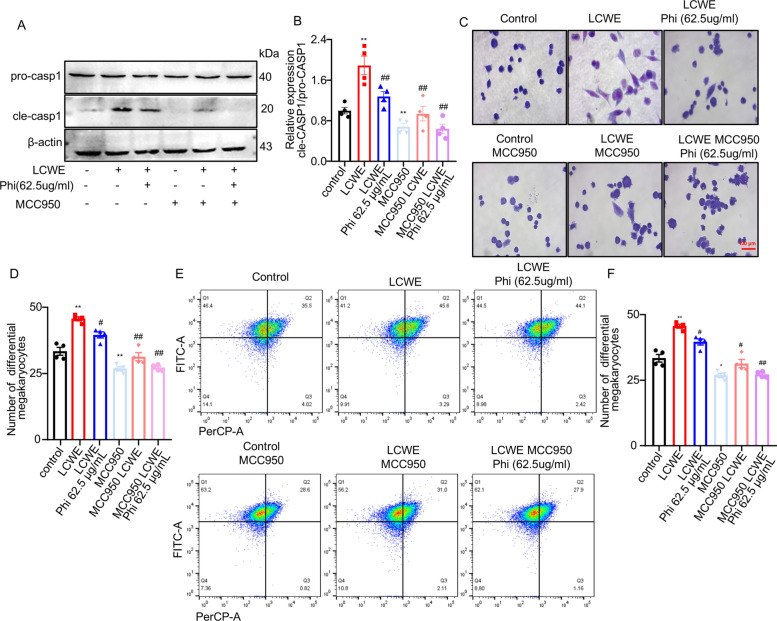
Inhibition of nlrp3 inflammasome assembly enhanced the effect of phillyrin on allevitated abnormal platelet production in MEG-01 cells. **A, B** Immunblot analysis of pro-caspase1 and cle-caspase1 level in MCC950-treated MEG-01 cells. **C, D** Representative image of MGG stain and count of differential megakaryocytes in MCC950-treated MEG-01 cells. **E, F** Representative flow cytometric images and quantification of differentiation MEG-01cell. n = 4 per group. Data are presented as mean ± SD. **P < 0.01 compared with control group, ^#^*P* < 0.05 versus LCWE group, ^##^*P* < 0.01 versus LCWE group

To further validate the inhibitory effect of phillyrin on the NLRP3 inflammasome in MEG-01 cells, NLRP3-specific inhibitor MCC950 were used to inhibit the activation of NLRP3 inflammasome. Western blot analysis revealed that the co-treatment of phillyrin and MCC950 resulted in an enhanced suppression of NLRP3 inflammasome activation, as evidenced by reduced levels of caspase-1 and cleaved caspase-1, compared to individual phillyrin treatments in MEG-01 cells (Fig. 6A, B). Subsequently, MGG staining also showed that the combined treatment of phillyrin and MCC950 caused less pseudopodia formation of MEG-01 compared with phillyrin intervention alone (Fig. 6C, D). Furthermore, the inhibitory effect on MEG-01 cell differentiation into platelet precursors, as assessed by flow cytometry, was further enhanced by the combination of phillyrin and MCC950, resulting in a more potent suppression than phillyrin monotherapy (Fig. 6E, F). Notably, MCC950-mediated inhibition of the NLRP3 inflammasome significantly suppressed megakaryocytic differentiation into platelet precursors. Collectively, these findings indicated that inhibition of NLRP3 inflammasome activation enhances the regulatory effect of phillyrin on platelet production in MEG-01 cells, further confirming the mechanism by which phillyrin modulates thrombopoiesis through targeting the NLRP3 inflammasome.

### Phillyrin inhibited IL-1β-induced expression of NF-E2 to impede platelet production

**Fig. 7:**
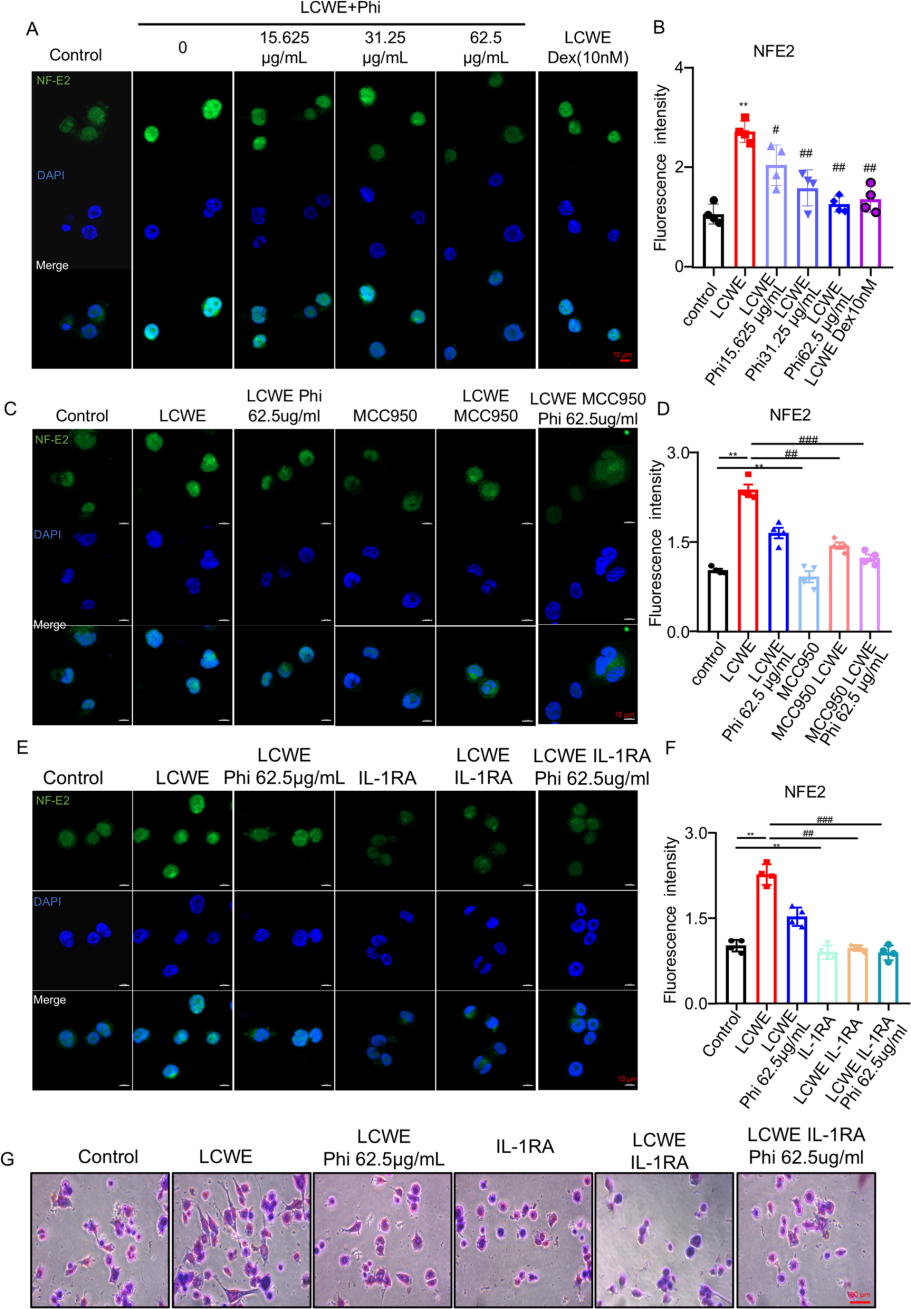
Phillyrin inhibited IL-1β–induced expression of NF-E2 to impede platelet production. **A, B** Representative confocal images and quantification of NF-E2 in MEG-01 cells. NF-E2 (green), DAPI (blue), scale bar: 10 μm. **C, D** Representative confocal images and quantification of NF-E2 in MCC950-treated MEG-01 cells. NF-E2 (green), DAPI (blue), scale bar: 10 μm. **E, F** Representative confocal images and quantification of NF-E2 in IL-1RA-treated MEG-01 cells. NF-E2 (green), DAPI (blue), scale bar: 10 μm. **E, F** Representative confocal images and quantification of NF-E2 in MEG-01 cells treated with IL-1β receptor antagonist. NF-E2 (green), DAPI (blue), scale bar: 10 μm. **G** Representative image of MGG stain of differential megakaryocytes in MEG-01 cells treated with IL-1β receptor antagonist. n = 4 per group. Data are presented as mean ± SD. **P < 0.01 compared with control group, ^#^*P* < 0.05 versus LCWE group, ^##^*P* < 0.01 versus LCWE group

To further delineate the pathway downstream of NLRP3/IL-1β inhibition, we assessed the effect of phillyrin on the IL-1β/NF-E2 signaling axis in megakaryocytes based on prior studies. Notably, NF-E2 expression was significantly upregulated by LCWE stimulation, while phillyrin treatment dose-dependently inhibited such upregulation (Fig. 7A, B). Furthermore, co-treatment of phillyrin and MCC950 also resulted in a more potent suppression of NF-E2 expression compared to individual phillyrin treatments in MEG-01 cells (Fig. 7C, D). Since IL-1β signals through the IL-1 receptor (IL-1R) to regulate NF-E2, MEG-01 cells were treated with the IL-1R antagonist IL-1RA to block receptor activation. Immunofluorescence analysis revealed that IL-1RA not only suppressed NF-E2 expression but also synergized with phillyrin to further inhibit its levels in MEG-01 cells (Fig. 7E, F). Consistent with this, MGG staining also showed that IL-1RA suppressed MEG-01 cells differentiation and markedly enhanced the inhibitory effect of phillyrin (Fig. 7G, H). Together, these data demonstrated that phillyrin inhibited the NLRP3/IL-1β pathway, thereby reducing NF-E2 expression and ultimately impeding megakaryocyte differentiation.

## Discussion

This study demonstrated the protective effects and underlying mechanisms of phillyrin on KD-promoted platelets generation and lung inflammation. LCWE were employed to induced mouse KD model and MEG-01 cell model. Platelet count, lung inflammation, MEG-01 cell differentiation and molecular mechanism based on NLRP3/IL-1β/NF-E2 pathway were detected. We found that (1) Phillyrin mitigated pulmonary inflammation induced by LCWE in mice by inhibiting MKs-mediated the production of platelet count in the lung. (2) Phillyrin inhibits MKs differentiation to secrete platelet via suppressed NLRP3/IL-1β/NF-E2 signaling pathway.

KD is a system vasculitis, primarily occurs in children less than 5 years old. If untreated, approximately 25% of children with Kawasaki disease develop coronary artery aneurysms, which may progress to myocardial infarction, rupture, or death [26]. Clinical observations showed that Kawasaki disease (KD) features thrombocytosis and pneumonia [27, 28]. Previous studies showed that increase of platelets count promotes the development of pneumonia, and depletion of platelets alleviated lung inflammation [9, 10]. Intraperitoneal injection of LCWE is a well-established experimental model for KD in rodents [29]. Therefore, both animal and cell experimental model were conducted via stimulating with LCWE in our study. Consistent with previous studies, significantly elevated platelet counts in the blood and lung tissue and induced severe pulmonary inflammation. Phillyrin treatment effectively alleviated these pathological alterations in a dose-dependent manner. While treatment with phillyrin remarkably reversed those symptoms in a dose-dependent manner (Fig. 1). Beyond their classical role in clot formation, platelets are among the first responders to sites of inflammation due to their rapid recruitment and abundance. Through interactions with the endothelium and immune cells, they actively mediate proinflammatory effects [30]. In this study, we further confirmed the therapeutical effects of phillyrin is through suppressing MKs-mediated platelets production in vitro. Our data indicated that phillyrin inhibited platelet production by suppressing the differentiation of MEG-01 megakaryocytic cells (Fig. 4). Furthermore, the finding that up to 8% of murine megakaryocytes (MKs) reside in the lungs provides an anatomical precedent for our results [31]. It supports the anti-inflammatory effect of phillyrin is mediated through the suppression of excessive platelet production within the lung.

To further investigate molecular mechanism underlying the therapeutic effect of phillyrin, we performed KEGG enrichment analysis on genes common to pneumonia, vasculitis, and thrombocytosis. The results highlighted a critical role for the NOD-like receptor protein and IL-1β signaling pathway in the pathogenesis of pulmonary thrombocytosis associated with KD (Fig. 2A–C). Emerging evidences revealed that LCWE promoted the activation of NLRP3 inflammasome, contributed to is IL-1β maturation and secretion, subsequently causesd lung injury [18, 32, 33]. Combining molecular docking with experimental validation, we identified phillyrin significantly inhibited megakaryocytic NLRP3/IL-1β signalling both in vivo and in vitro (Figs. 2D–H, 5). This is consistent with prior studies establishing NLRP3’s essential role in MKs-mediated platelet production and activation [34]. Moreover, our findings aligned with existing evidence that phillyrin exerts protective effects against pulmonary inflammation by inhibiting NLRP3 inflammasome activation [20, 35]. In addition, our results indicated that NLRP3^−/−^ mice showed a significant, yet physiologically normal, decrease in platelet counts compared to wild-type mice. Notably, both knockout of NLRP3 in mice and pharmacological inhibition of its activation in MEG-01 cells significantly enhanced the protective effects of phillyrin (Figs. 3, 6). Functional ablation of NLRP3 knockout were validated, as evidenced by the significant reduction in cleaved caspase-1 and mature IL-1β. Nevertheless, whether phillyrin exerts its inhibitory effects by modulating NLRP3 expression-related pathway requires further investigation. In addition, IL-1β secretion was also mediated by other NLR inflamasomes, including NLRP1, AIM2, NLRC4 [36], we also found the inhibitory effect of phillyrin on these inflammasome (supplemental figure), its inhibitory effect on NLRP3 in megakaryocytes was the most pronounced.

NLRP3 inflammasome-induced secretion of IL-1β is crucial in controlling the deferentiation of MKs, the generation and activation in platelet [37, 38]. Previous studies have established NF-E2 as a pivotal transcription factor that regulates MK maturation and platelet production, whose expression is markedly upregulated in MKs in response to elevated IL-1β levels [39, 40]. NF‐E2 is the prime regulator of terminal MK differentiation and platelet release, it regulates the expression of NOTCH1, 17‐*β* estradiol, *β*1‐tubulin in MKs, which were essential for the differential and formation of platelet discoid [41, 42]. Therefore, we investigated whether phillyrin suppresses NLRP3/IL-1β signaling, thereby interfering with IL-1β receptor binding and consequently impeding NF-E2-driven platelet production. Our results demonstrated that phillyrin significantly reduced LCWE-induced NF-E2 expression. Moreover, both inactivation of the NLRP3 inflammasome and pharmacological antagonism of IL-1R similarly downregulated NF-E2 expression (Fig. 7), consistent with the inhibitory effect of phillyrin on platelet production. Collectively, these findings confirmed that phillyrin attenuated LCWE-induced platelet production and lung inflammation by inhibiting the NLRP3/IL-1β/NF-E2 signaling pathway.

## Conclusions

Phillyrin significantly alleviated LCWE-induced pulmonary inflammation by suppressing megakaryocyte-derived platelet production. Mechanistically, phillyrin inhibited the activation of the NLRP3 inflammasome, thereby impeding caspase-1 maturation and IL-1β secretion. The blockade of IL-1β binding to its receptor consequently downregulated the expression of NF-E2, a pivotal transcription factor regulating platelet production (see Graphical abstract). Collectively, our findings elucidated the therapeutic mechanism of phillyrin against KD-induced lung injury and provided a scientific rationale for its clinical application in treating Kawasaki disease and associated thrombocytosis.

## Supplementary Information

Additional file 1

Additional file 2

Additional file 3

## Data Availability

The data that support the findings of this study are available from the corresponding author upon reasonable request.

## Abbreviations

IF: Immunofluorescence
IHC: Immunohistochemistry
IL-1β: Interleukin-1β
LCWE: Lactobacillus casei cell wall extract
KD: Kawasaki disease
MKs: Megakaryocytes
NLRP3: NOD-like receptor thermal protein domain associated protein 3
MGG: May-Grünwald and Giemsa
Phi: Phillyrin
PLT: Platelet count
WBC: White blood cell
