# Supplementary figures and images for: Phillyrin alleviates Kawasaki disease-induced lung inflammation by inhibiting platelet production through the NLRP3/IL-1β/NF-E2 signaling pathway
Source: Chin Med. 2026 Mar 7;21:80. doi: 10.1186/s13020-026-01348-6 (PMC12967014; doi:10.1186/s13020-026-01348-6)

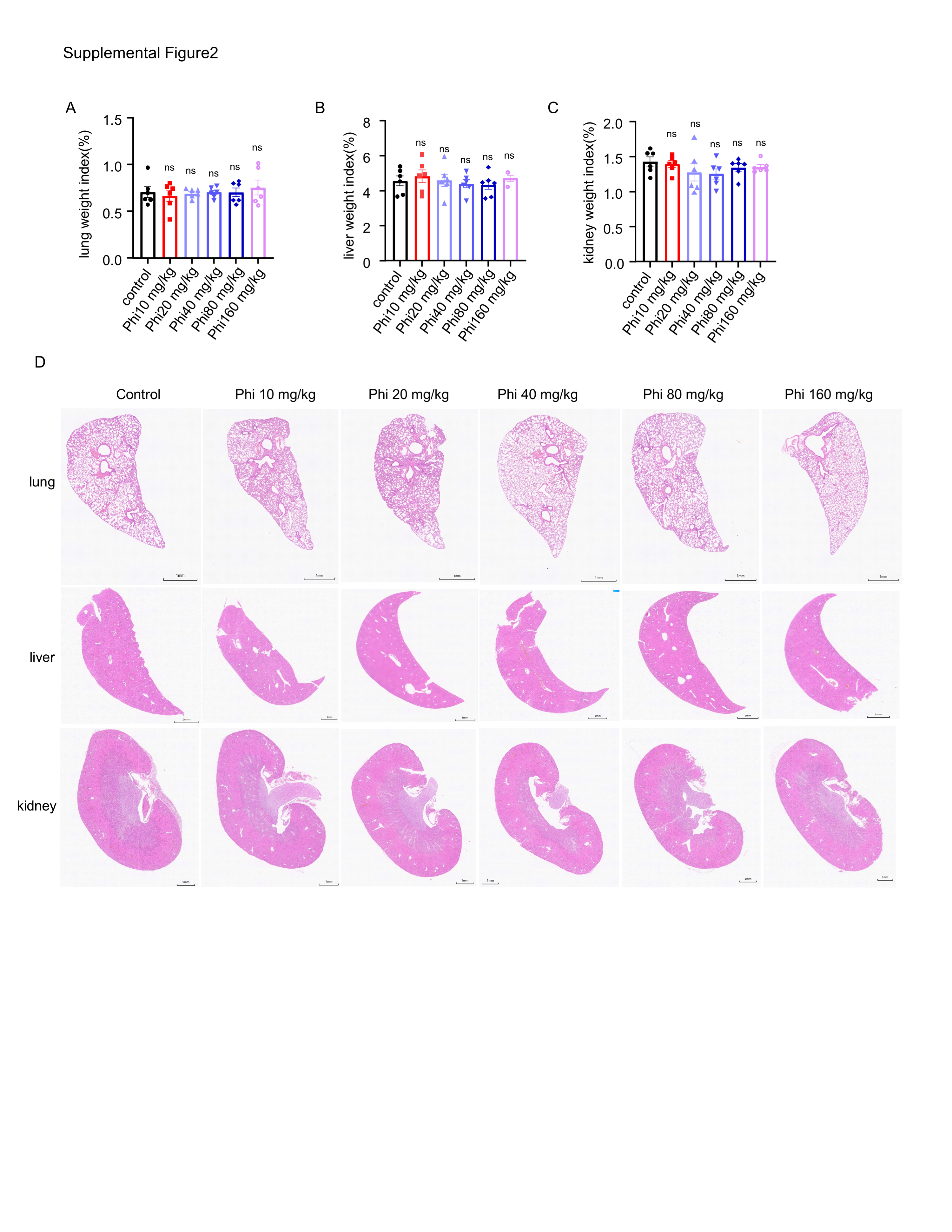

Supplement: Supplementary file 1 — Additional file 1 [file 13020_2026_1348_MOESM1_ESM.tif]

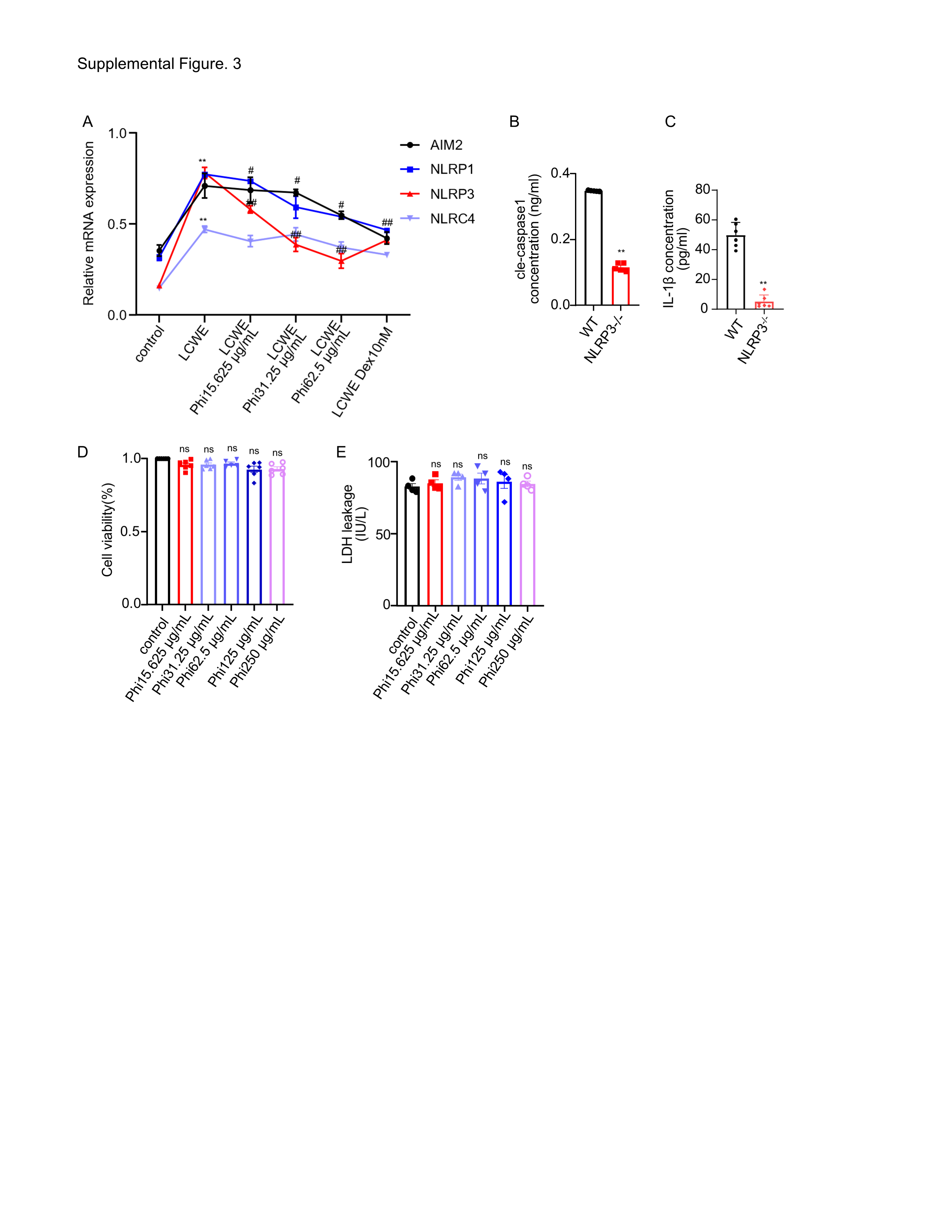

Supplement: Supplementary file 2 — Additional file 2 [file 13020_2026_1348_MOESM2_ESM.tif]

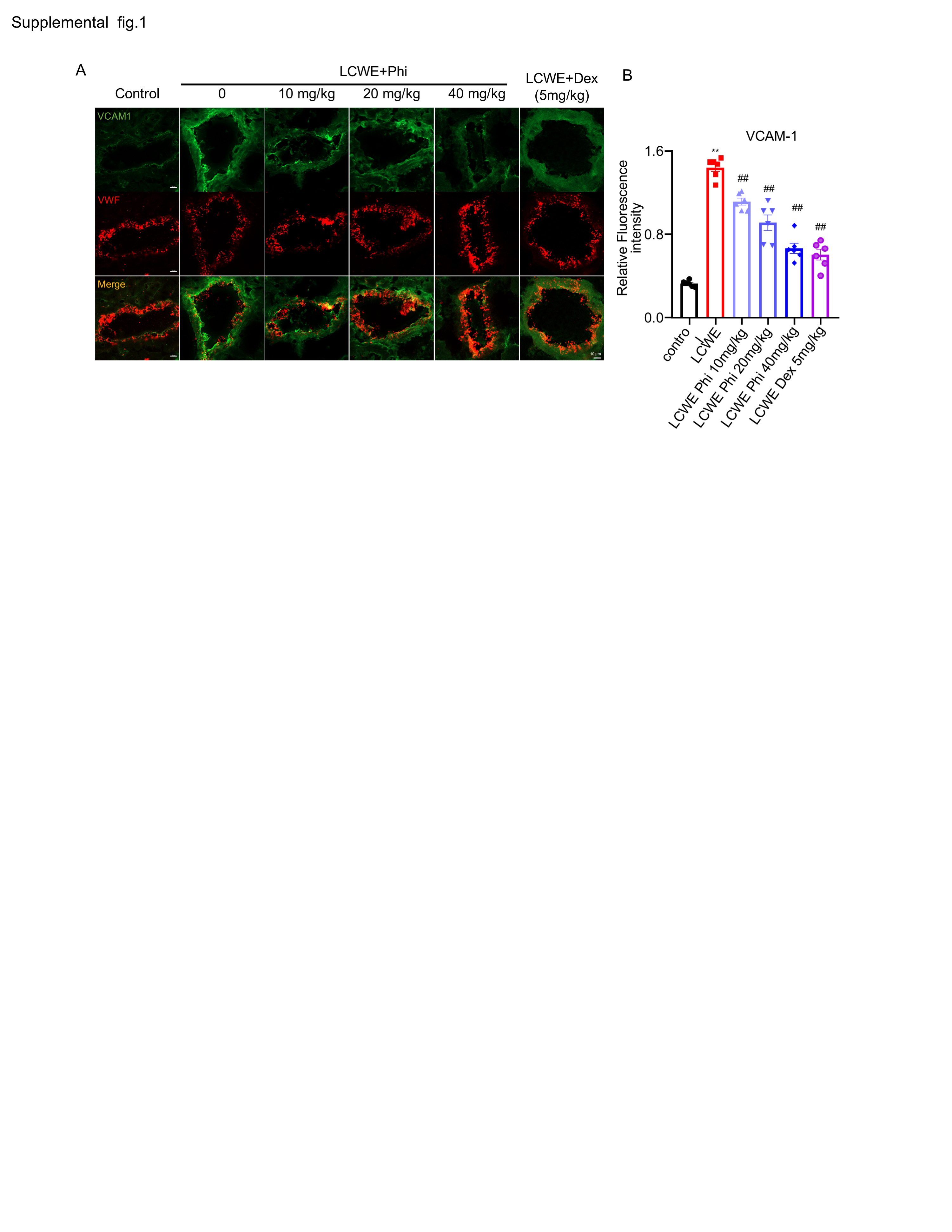

Supplement: Supplementary file 3 — Additional file 3 [file 13020_2026_1348_MOESM3_ESM.tif]
